# Non-Coding RNAs As Transcriptional Regulators In Eukaryotes

**Published:** 2017

**Authors:** O. Y. Burenina, T. S. Oretskaya, E. A. Kubareva

**Affiliations:** Skolkovo Institute of Science and Technology, Nobel Str. 3, Moscow, 143026, Russia; Lomonosov Moscow State University, Chemistry Department, Leninskie Gory 1, bld. 3, Moscow, 119991 , Russia; Belozersky Institute of Physico-Chemical Biology, Lomonosov Moscow State University, Leninskie Gory 1, bld. 40, Moscow, 119991, Russia

**Keywords:** noncoding RNAs, RNA polymerase, transcription regulation

## Abstract

Non-coding RNAs up to 1,000 nucleotides in length are widespread in eukaryotes and fulfil various regulatory functions, in particular during chromatin remodeling and cell proliferation. These RNAs are not translated into proteins: thus, they are non-coding RNAs (ncRNAs). The present review describes the eukaryotic ncRNAs involved in transcription regulation, first and foremost, targeting RNA polymerase II (RNAP II) and/or its major proteinaceous transcription factors. The current state of knowledge concerning the regulatory functions of SRA and TAR RNA, 7SK and U1 snRNA, GAS5 and DHFR RNA is summarized herein. Special attention is given to murine B1 and B2 RNAs and human Alu RNA, due to their ability to bind the active site of RNAP II. Discovery of bacterial analogs of the eukaryotic small ncRNAs involved in transcription regulation, such as 6S RNAs, suggests that they possess a common evolutionary origin.

## INTRODUCTION

According to transcriptome analysis, only 1.5% of the total amount of RNAs in eukaryotic cells encodes proteins, while other transcripts are non-coding (ncRNAs). Apparently, the ”repertoire” of genes that encode proteins has remained relatively static in the course of evolution, and the number of ncRNA genes has increased when proceeding to more complex organisms. Ribosomal, transfer, small nuclear, and small nucleolar ncRNAs, which are constantly expressed in the cells, are conventionally classified as housekeeping ncRNAs, by analogy with the name of the most important cellular genes [1]. However, the majority of ncRNAs fulfil regulatory functions and participate in the equally important and often conversely directed molecular processes such as DNA demethylation and imprinting, activation and repression of gene transcription, as well as chromatin remodeling, RNA interference, and alternative splicing [2-4]. The level of synthesis of many ncRNAs varies under different stress conditions, during cancer and neurologic diseases [5, 6]. ncRNAs play a major role in cell differentiation [7]. Taking into account the fact that this is only a small part of the currently known properties and functions of ncRNAs, one can assume that their contribution to the maintenance of a normal functioning of the cell is no less significant than the contribution of protein factors.

Usually ncRNAs are classified into short (~20–30 nt), which include microRNAs (miRs), small interfering (siRNAs), and PIWI-interacting RNAs (P-element-induced wimpy testis, piRNA) [8]; small ncRNAs up to 200 nt; and long ncRNAs ( > 200 nt). Among small ncRNAs, promoter-associated RNAs (paRNAs) are the most well-known, although this class includes representatives of various lengths [9]. The term “long non-coding RNAs” (lncRNAs) is widely used for the transcripts that are several thousand nucleotides in length and belong to long intergenic ncRNAs (lincRNAs) and enhancer RNAs (eRNAs) [10]. However, there are also extremely lengthy ncRNAs, consisting of several hundred thousand nucleotides, such as very long intergenic ncRNAs (vlincRNAs) and macroRNAs [11].

**Fig. 1:**
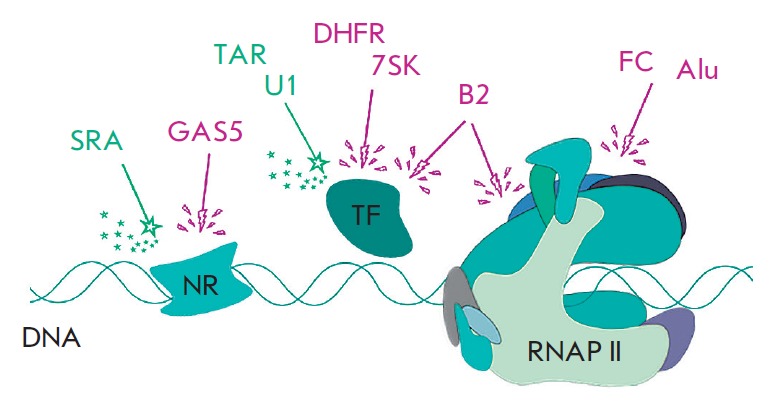
The best known ncRNAs acting as transcriptional activators (green) or inhibitors (purple) via interactions with RNAP II and/or its general transcription factors (TF) or with other regulatory proteins, in particular nuclear receptors (NR).

Considering the diversity of the classes and functions of ncRNAs, it is no surprise that many of them are involved in the regulation of transcription in eukaryotes. This occurs primarily through various epigenetic mechanisms; in particular, chromatin remodeling (this area of ncRNA functioning has been studied much better than others) [12, 13]. The well-known examples of such ncRNAs include XIST RNA (X-inactive specific transcript), roX RNA, HOTAIR (Hox transcript antisense intergenic RNA); enhancer RNAs NRIP1, GREB1, KLK; and NEAT1 RNA (nuclear enriched abundant transcript 1), which is responsible for paraspeckle formation in tumor cell nuclei. Regulation of co-transcriptional splicing involves MALAT1 RNA (metastasis-associated lung adenocarcinoma transcript 1) and H19 RNA, which serve as therapeutic targets for various diseases, including cancer [14]. In addition, there are ncRNAs that interact with RNA polymerase II (RNAP II) or the transcription factors incorporated in the preinitiating complex (PIC) or elongation complex. The latter include 7SK small nuclear RNA (snRNA) and TAR RNA, which regulate the activity of the transcription elongation factor P-TEFb; U1 snRNA, which interacts with the initiation factor TFIIF; SRA RNA that activates steroid receptors, and some others (*Fig. 1*). These ncRNAs are involved in complex multistep regulatory mechanisms and usually interact with cascades of proteins, indirectly affecting a transcription process. On the contrary, the murine B1 and B2 RNAs and human Alu RNA encoded by mobile genetic elements (SINE) are able to directly bind RNAP II [15]. To date the X-ray data of their complexes with the enzyme has not been obtained. FC RNA, the synthetic aptamer consisting of two short hairpins, is the only ncRNA whose complex with RNAP II has been solved by X-ray analysis [16]. Since the secondary structure of almost all of the aforementioned regulatory ncRNAs includes short hairpin elements, which interact with the active site of RNAP II, they are often regarded as aptamers for the enzyme [17].

According to the conventional criteria, TAR RNA, B1 RNA, B2 RNA, and U1 snRNA should be classified as small ncRNAs, while Alu RNA, 7SK snRNA, DHFR RNA, SRA RNA, and GAS5 RNA are lncRNAs. The specific properties and functions of each of these ncRNAs are discussed in detail in this review. The structural features of the interaction between FC RNA and RNAP II are comprehensively described in [16].

## REGULATORY RNAs ENCODED BY GENETIC ELEMENTS OF THE SINE FAMILY

SINE (short interspersed elements) are retrotransposons 80 to 500 bp in length located randomly in the genome of higher eukaryotes. The nucleotide sequences of SINE characterized by 65–90% similarity are clustered into families, and the number of homologous SINE can vary from 103 to 106 copies per cell [18]. Historically, SINE had been considered as “genetic garbage” used to establish phylogenetic relationships and study speciation in mammals until it was found that transcription of SINE-“genes” is activated in cells in response to heat shock [19]. It is believed that this is due to the increased accessibility of SINE for transcription in chromatin remodeling and activation of the transcription factor TFIIIC binding to the promoter regions of SINE. As it turned out, SINE are involved in the regulation of gene expression, localization of mRNA, and they can act as enhancers or mobile promoters for RNAP II [20]. To date, it is known that SINE do not encode proteins and are transcribed by RNAP III into the corresponding SINE RNAs. Unexpectedly, it was discovered that some SINE RNAs are capable of binding RNAP II and inhibiting transcription. The main results were obtained for murine B1 and B2 RNAs and human Alu RNA [14, 21]. Exposure to UV and γ-radiation, viral infections, ethanol, antibiotics and anticancer drugs induces an increase in the expression level of these ncRNAs in cells [14]. These data certainly suggest an important functional role for B1, B2, and Alu RNAs in cell life.

**Human Alu RNA and murine B1 RNA**

**Fig. 2:**
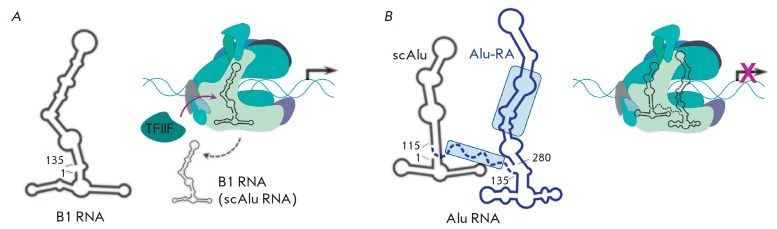
Scheme of the functioning of murine B1 RNA (**A**) and human Alu RNA (**B**). Secondary structures of ncRNAs are schematically shown on the left. Alu RNA structural elements responsible for transcription inhibition are in light-blue frames, the functional domain (Alu-RA) is colored in blue, the A-rich linker is shown by a dash line. A schematic view of interactions between RNAP II and B1, scAlu, or Alu RNA is shown on the right. Transcription is indicated by a black arrow. B1 and scAlu RNA are displaced by TFIIF from their complexes with RNAP II, thus, they are unable to inhibit transcription, in contrast to Alu RNA.

SINE element Alu was named due to the presence of recognition sites of restriction endonuclease from *Arthrobacter luteus *(R.AluI). The human genome contains more than 1 million copies of Alu encoding Alu RNAs, which accounts for about 10.6% of nuclear DNA. B1 RNA-encoding SINE are more rare in the murine genome, less than 550,000 per cell. Both of these RNAs belong to the family of retroelements of small cytoplasmic 7SL RNA [22] and possess a similar secondary structure (*Fig. 2*). The full-length Alu RNA, sized ~280 nt in length, is a tandem repeat of two B1-like elements connected by a 20-nt A-rich linker. Alu RNA processing produces scAlu RNA of 118 nt in length, which is localized in the cytoplasm and is a complete analog of murine B1 RNA (*Fig. 2*) [23]. Alu RNA has an unusual shape, hence its structured parts were named “left arm” (identical to scAlu RNA) and “right arm” (Alu-RA, 135–280 nt of Alu RNA). Each Alu RNA domain can bind one RNAP II molecule, but only interaction of Alu-RA (or full-sized Alu RNA) with the enzyme results in the inhibition of transcription. Murine B1 RNA, despite its high affinity to RNAP II, cannot affect transcription (*Fig. 2A*), although chimeric RNA consisting of B1 RNA and Alu-RA has all the properties of the full-size Alu RNA [24, 25].

Besides the two RNAP binding domains located in the “left arm” and “right arm,” Alu RNA has two domains disposed in the central region of the “right arm” and in the A-rich linker which are responsible for transcription inhibition (*Fig. 2B*). Correspondingly, B1 RNA and scAlu RNA possess only a RNAP binding domain. According to cryoelectron microscopy, both Alu and B1 RNA interact with the “clamp” domain of RNAP II near the active site of the enzyme [26]. So how does the transcription occur in case of non-functional B1 and scAlu RNAs? It has been shown that RNAP II is released from its complexes with B1 and scAlu RNA under the action of the transcription factor TFIIF, causing a dissociation of these ncRNAs from PIC, while Alu RNA remains bound to the polymerase (*Fig. 2*). At the same time, no direct contact between TFIIF and B1 or scAlu RNA was detected [27]. The disruption of RNA-protein contacts is likely to occur during conformational changes in RNAP caused by TFIIF binding. Since *in vivo *TFIIF is usually associated with RNAP II prior to PIC assembly on the promoter, “useless” binding of non-regulatory ncRNAs (having no effect on the transcriptional activity of RNAP) probably does not occur.

The precise mechanism of interaction between Alu RNA and PIC is not fully understood. Transcription inhibition was observed *in vitro* only when Alu RNA was added prior to the initiation of transcription from the promoter, although the efficiency of abortive transcript synthesis in the presence of Alu RNA was ~ tenfold lower. At the same time, electrophoretic mobility shift assay has shown that Alu RNA comigrates together with the DNA as a part of PIC RNAP II [23]. Thus, transcription is inhibited not owing to competition with DNA, but rather as a result of altered enzyme activity due to the formation of specific ncRNA-protein contacts. However, Alu RNA cannot stop active transcription and fulfils its functions before the initiation step.

**Murine B2 RNA**

B2 RNA is transcribed by RNAP III in the presence of TFIIIB and TFIIIC factors from the respective B2 SINE (belonging to the tRNA^Ala^ retroelement family), whose number is estimated to be about 10^5^ copies per cell [28]. This RNA can be isolated in complex with RNAP II during immunoprecipitation of the nuclear extracts of cells exposed to heat shock [29], and it is capable of inhibiting transcription *in vitro *[25]. Knockdown of B2 RNA in murine cells leads to increased expression of actin and hexokinase II, whereas their genes are repressed under heat shock conditions [24]. Increase in the amount of B2 RNA has been observed during cell response to various stress factors, as well as in embryonic and tumor cells [30]. Thus, the important role of this ncRNA as a transcription inhibitor is undoubted. Unfortunately, there is only scarce data on the nature of B2 RNA functioning *in vivo*, while, the mechanism of action of this ncRNA has been studied in detail.

**Fig. 3:**
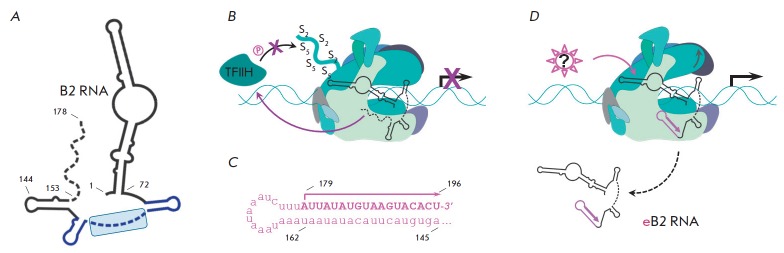
Scheme of the functioning of murine B2 RNA. (**A**) Simplified view of the B2 RNA secondary structure. Disordered parts are shown by a dash line; functional part – by a blue line; inhibitory domain is in a light-blue frame. (**B**) B2 RNA prevents initiation of transcription by “switching off” TFIIH kinase activity. The Ser2 and Ser5 amino acid residues are marked as “S2” and “S5,” phosphorylation – as “P” in a circle. (**C**) The sequence of the 3’-end fragment (145–178 nt) of B2 RNA extended by additional 18 nt [34]. (**D**) Elongation of B2 RNA 3’-end (eB2 RNA) leads to the formation of a new hairpin (pink), which causes conformational changes in RNAP II (shown by a grey arrow) and dissociation of B2 RNA. Transcription on a DNA template is indicated by a black arrow, transcription on a B2 RNA template – by a pink arrow.

Murine cells contain at least four variants of B2 transcripts of different lengths: ~150, ~180, ~240 and ~500 nt. The two longest variants are very stable (τ_1/2_ = 60 min) due to polyadenylation, whereas the degradation time of a 180 nt-long transcript is just 3–4 minutes. The shortest 150-nt variant of B2 RNA is more stable and characterized by a τ_1/2_ value of 20 min [31]. In 2004, the secondary structure of the ~180 nt-long transcript was determined [25], consisting of three nominal fragments (*Fig 3A*): (1) a long double-stranded region (1–72 nt) with unwound moiety in the center; (2) a poorly structured region (73–153 nt) containing three small hairpins, and (3) a short 3'-terminal unstructured AU-rich region (154–178 nt) which is conserved in all SINE.

Footprinting studies have shown that RNAP II binds to the least structured part of the molecule (73–155 nt), and a 5'-terminal hairpin is required neither for B2 RNA binding to RNAP II nor for transcription repression. Analysis of various deletion mutants of B2 RNA has determined the 51 nt-long region (81–131 nt) which directly interacts with RNAP II and inhibits transcription *in vitro *with the same efficiency as full-length B2 RNA [31]. Notably, the unstructured region of B2 RNA (99–115 nt) flanked by two hairpins plays the most important role in transcription inhibition. Removal of any of them leads to a loss of inhibitory activity of a fully functional deletion derivative of B2 RNA (81–131 nt). At the same time, absence of these hairpins in full-length B2 RNA has no effect on its properties. Besides, all these deletion mutants of B2 RNA demonstrated specific binding to RNAP during PIC assembly on the promoter [31, 32]. Therefore, transcriptional repression requires correct positioning of a single-stranded region (99–115 nt) of B2 RNA complexed with RNAP II, which, apparently, can be achieved by any of the present hairpins.

The similarity of the structural organization of B2 RNA and Alu RNA indicates that, besides the active site (whose blocking leads to global inhibition of mRNA synthesis), RNAP II contains an additional docking site highly specific to ncRNAs. As in the case of Alu RNA, RNAP II forms a ternary complex *in vitro *with B2 RNA and the promoter simultaneously [25]. Therefore, B2 RNA can also bind to RNAP after the formation of a stable complex with the promoter and inhibit transcription at the stage of initiation. This disables not only the synthesis of full-length mRNA, but also abortive transcripts. Experiments on crosslinking and footprinting of PIC associated with B2 RNA have shown that this ncRNA hinders proper coordination of the promoter in the active site of the polymerase and, thereby, switches PIC into its inert form. In fact, B2 RNA alters the conformation of the “closed” RNAP complex and prevents its conversion into an “open” complex and, all the more, into an initiation complex. At the same time, all factors associated with PIC, including TBP and TFIIB, remain bound to the promoter and hold the complex on the DNA [25].

Let us recall that murine cells also express B1 RNA binding to RNAP II, but are incapable of inhibiting transcription. B1 RNA possesses affinity to polymerase comparable to that of B2 RNA and can displace B2 RNA from PIC. Therefore, B1 RNA must prevent the functioning of B2 RNA. However, it has been shown *in vitro *that B2 RNA can inhibit transcription even in the case when PIC had been previously bound to B1 RNA [27]. The mechanisms of competition between these two ncRNAs *in vivo *have not been established. Since human Alu RNA also has a non-functional analogue, it can be assumed that these inactive ncRNAs, B1 and scAlu RNA, in certain circumstances may replace B2 and Alu RNA, respectively, and re-stimulate transcription.

Interestingly, in addition to direct “physical” RNAP II active site blocking, B2 RNA specifically inhibits the kinase activity of the TFIIH transcription factor (*Fig. 3B*). TFIIH contains cyclin-dependent kinase 7 (CDK7), which, under normal conditions, phosphorylates serine residues within the heptapeptide repeats YSPTSPS (mainly Ser5) at the C-terminal domain (CTD) of the large subunit (Rpb1) of RNAP II. Modification of Ser2 and Ser5 at the CTD of Rpb1 is extremely important for transcription. It occurs at various stages of transcription: the domain is not phosphorylated in the initiation complex and, on the contrary, hyperphosphorylated during transcription elongation [33]. Thus, B2 RNA not only creates conformational constraint in RNAP II itself, but also disables the proceeding to the elongation stage, affecting the functioning of the transcription factor. Although TFIIH is not the primary target of B2 RNA and its repression is likely due to the interaction between B2 RNA and PIC, this is a unique phenomenon for ncRNAs.

The more surprising fact is that B2 RNA can promote self-elongation in a complex with RNAP II [34]. The enzyme uses the 3'-end of the B2 RNA molecule as a template for *de novo *transcription and synthesizes 18 additional nucleotide residues, forming a stable extended hairpin (*Fig. 3 C, D*). Elongation of the B2 RNA leads to the dissociation of the molecule from PIC and, apparently, enables reversibility of inhibition. The released extended B2 RNA undergoes degradation. An analysis of computer modeling data suggests that the elongation of the B2 RNA strand (or any other RNA located in the active site of the polymerase) should lead to the partial opening of the “clamp” domain of RNAP and, as a consequence, the weakening of ligand binding to the enzyme. In fact, a newly formed structural element of the elongated B2 RNA “extrudes” the molecule from PIC. Notably, B2 RNA elongation was observed *in vitro *only after treatment of the B2 RNA-RNAP II complex with the cell extract [34]. It is believed that the RNA-dependent transcription of RNAP II is initiated by a protein factor whose nature is yet unknown.

Since most RNA polymerases are DNA-dependent (except for retroviral RNAPs), elongation of B2 RNA is a kind of exception to the rule, since the enzyme modifies its substrate specificity. To date, there are only a few such examples, which are also related to the functioning of ncRNAs. For instance, the same mechanism is used by the hepatitis delta virus and plant viroids to replicate their own genome. These pathogenic circular ncRNAs lack inherent RNA polymerases and use host cell RNAPs, reprogramming them for RNA synthesis on RNA templates [35]. This phenomenon might be more clearly exemplified by prokaryotic 6S RNA, which inhibits transcription due to interaction with RNAP, similarly to B2 RNA. Under certain conditions, bacterial RNAP can synthesize short transcripts (pRNAs) up to 30 nt in length on 6S RNA as a template. In this case, the enzyme dissociates from the complex with 6S RNA and resumes transcription from gene promoters [36]. Therefore, despite the huge differences in the transcription processes in prokaryotes and eukaryotes, there is undoubted similarity between the functioning of bacterial 6S RNA and murine B2 RNA.

## NON-CODING RNAs REGULATING THE ACTIVITY OF GENERAL TRANSCRIPTION FACTORS

**U1 snRNA**

**Fig. 4:**
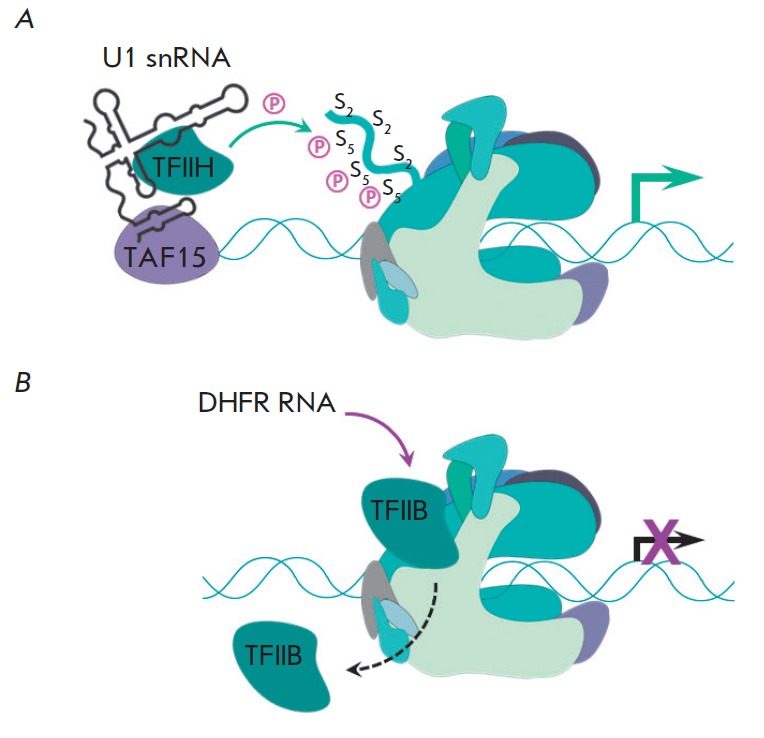
Scheme of the functioning of U1 snRNA (**A**) and DHFR ncRNA (**B**). The simplified secondary structure of U1 snRNA is adapted from [37]. There is no structural data for DHFR ncRNA. U1 snRNA activates transcription (shown by green arrow) by stimulating the TFIIH-dependent phosphorylation of RNAP II Rpb1 CTD. DHFR ncRNA inhibits transcription by displacing the TFIIB transcription factor from PIC.

U1 snRNA is one of the five major snRNAs forming the core of the spliceosome. Human U1 snRNA, 164 nt in length, is associated with U1-A, U1-C, and U1-70k proteins, as well as with eight Sm proteins, together forming the U1 snRNP complex (~245 kDa). The main function of U1 snRNP is to recognize pre-mRNA at the first (initial) stage of spliceosome assembly, which occurs due to complementary interactions between the 5'- end region of U1 snRNP and the intron splice site [37]. However, in addition to its primary role, U1 snRNA can interact with cyclin H (CycH) as a part of TFIIH, which in turn leads to an increased kinase activity of another subunit of this factor, CDK7 (*Fig. 4A*). Studies of *in vitro *transcription showed that the presence of U1 snRNA in the reaction mixture increases the rate of formation of the first phosphodiester bond and that the efficiency of transcription initiation increases more than tenfold. Furthermore, U1 snRNA stimulates abortive initiation and re-initiation of transcription from the promoter preceding the 5'-terminal splicing site [38]. Besides TFIIH, U1 snRNA may interact with other transcription factor – TAF15 – associated with TFIID in PIC and presumably involved in the elongation step [39]. For all intent and purposes, U1 snRNA activates the transcription process unlike the other aforementioned regulatory ncRNAs.

**DHFR ncRNA**

The *DHFR* gene encodes dihydrofolate reductase, one of the key enzymes of folate metabolism. About 99% of DHFR mRNAs are transcribed from the major promoter and contain six exons. During serum starvation and cell growth retardation, an alternate promoter located ~450 nt upstream from the main transcription initiation site is activated. Early transcription termination at the second intron results in the formation of a short product from the minor promoter, DHFR ncRNA, whose length varies from 800 nt to 2–3 thousand nt [40]. The functional part of the molecule is likely to be a ~400 nt-long fragment complementary to the promoter region of its own gene and containing long poly-(dG)-sequences. The latter are involved in the formation of an H-shaped purine-purine-pyrimidine triplex between DHFR ncRNA and the promoter that impedes PIC assembly [41]. Thus, DHFR ncRNA belongs to the class of promoter-associated ncRNAs [9]. Above that, it can interact with the transcription factor TFIIB incorporated in PIC, resulting in its dissociation [42]. Since TFIIB binding to the promoter is a key stage of PIC assembly and DHFR ncRNAs totally prevents this process, transcription is inhibited. It is yet unknown which region of DHFR ncRNA is responsible for the interaction with TFIIB, as well as the details of the processing of this ncRNA.

**7SK and TAR RNA**

Human 7SK snRNA and TAR RNA of HIV are probably the most well-known eukaryotic ncRNAs involved in the regulation of transcription elongation. Both of these ncRNAs act as platforms for the assembly of protein associates, modulating the activity of the RNAP II elongation complex, and they interact with the factor P-TEFb [43-45].

**Fig. 5:**
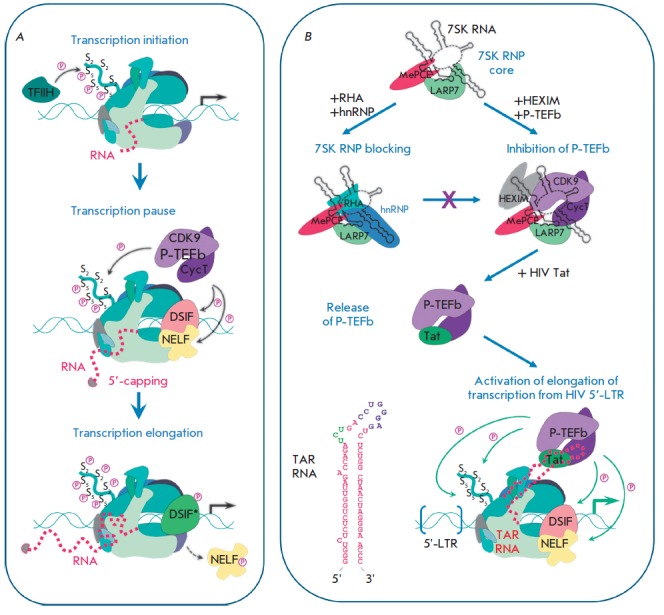
Transcription regulation involving human 7SK snRNA and HIV TAR RNA. (**A**) General steps of transcription conducted by RNAP II [46]. For transcription initiation, TFIIH phosphorylates Ser5 residues in RNAP Rpb1 CTD. The enzyme stops after the synthesis of a small transcript and the negative elongation factors NELF and DSIF bind to RNAP, resulting in transcription pause. After 5’-capping of a nascent RNA strand transcription restarts: DNA-binding proteins attract the P-TEFb factor, which further phosphorylates RNAP Rpb1 CTD and the factors NELF and DSIF. The latter turns into a transcription activator (DSIF*, shown in green), and modified NELF dissociates from the complex, that enables RNAP to proceed to the transcription elongation step. (**B**) Assembly of alternative protein complexes on 7SK snRNA. Binding of RHA and hnRNP to the 7SK snRNP core prevents the inhibition of P-TEFb. The HIV protein Tat can displace P-TEFb from 7SK snRNP and attract it to RNAP, arrested near the transcription start site. TAR RNA interacts with Tat and CycT and activates the kinase activity of P-TEFb, resulting in hyperphosphorylation of RNAP Rpb1 CTD and NELF/DSIF, followed by elongation of a viral transcript.

P-TEFb is a key transcription factor that stimulates the proceeding of RNAP II arrested at the promoter (the so-called transcription pause required for 5'-capping of the nascent mRNA strand) to the activation of elongation. P-TEFb consists of cyclin-dependent kinase 9 (CDK9) and cyclin T1 or its analogs, CycT1b and CycT2b (hereinafter CycT). Its main function includes phosphorylation of Ser2 in the CTD of Rpb1 RNAP II, as well as the transcription repressors NELF and DSIF [46] *(Fig. 5A)*. P-TEFb is attracted to the polymerase by various DNA-binding proteins, first of all Brd4, but also by some general transcriptional factors, such as NF- κB, HSF, p53, c-Myc, etc. After overcoming the pause stage, P-TEFb binds several other proteins, forming a super elongation complex (SEC) of RNAP II [47].

In the absence of P-TEFb, RNAP II can transcribe only short 5'-terminal sequences of pre-mRNA; i.e., this factor is required for the synthesis of most cellular mRNAs. When interacting with P-TEFb, 7SK snRNA inhibits its activity, which is an important regulatory mechanism of gene expression in eukaryotic cells. On the other hand, the release of P-TEFb from its complex with 7SK snRNA may serve as a signal for cell growth and proliferation [48]. On the contrary, HIV TAR RNA activates P-TEFb, which facilitates the initiation of transcription of the 5'-end of the viral promoter (5'- LTR) [45]. This review describes only the main features of these ncRNAs and their functioning principles.

7SK snRNA is 332 nt in length and consists of four main long hairpin structures connected by disordered areas and additional small hairpins. Although the human genome contains hundreds of 7SK snRNA pseudogenes, this RNA (~2 × 10^5^ copies per cell) is transcribed by RNAP III from a single genuine gene located on the sixth chromosome. The nucleotide sequence of this gene is highly conserved in vertebrates [49]. During post-transcriptional modifications, nucleases cleave 1–3 nt from the 3'-end of 7SK RNA, before adenylation occurs, resulting in three different isoforms of 7SK snRNA present in the cell: 330, 331 and 332 nt in length, of which 331-mer is the most stable. In addition, 7SK snRNA is capped at the 5'-end: methyltransferase MePCE methylates the γ-phosphate group of the 5'-terminal guanosine residue. This process is not characteristic of transcripts synthesized by RNAP III, and it has been previously described only for U6 and 7SK snRNAs [50].

Approximately 90% of 7SK snRNA in the cell is bound to MePCE and, together with the LARP7 protein, forms a so-called core of the ribonucleoprotein complex, 7SK snRNP *(Fig. 5B)*. MePCE and LARP7 also interact with each other, further stabilizing snRNP; in this state, 7SK RNA is protected from degradation. The complex further binds the HEXIM protein in the form of a dimer consisting of alternative HEXIM1 and/or HEXIM2 paralogs. The arginine-rich RNA-binding domain (ARM) of HEXIM binds the 5'-terminal hairpin of 7SK snRNA, resulting in conformational change in the protein, so that it can interact with CycT of P-TEFb. Additionally, the C-terminal domain of LARP7 binds to CDK9, providing a stable structure of the whole complex. Apparently, 7SK RNA is also involved in the formation of contacts with P-TEFb. As a result, the factor loses its kinase activity, which prevents it from promoting transcription elongation [48, 51, 52].

However, not all P-TEFb molecules are bound to 7SK snRNP. A wealth of experimental data shows that there is continuous equilibrium between the free and bound forms of P-TEFb in the cell nucleus, which is controlled through various signaling pathways. For example, a number of heterogeneous nuclear ribonucleoproteins (hnRNPs) and RNA helicase A (RHA) block access to P-TEFb through binding to the 7SK snRNP core *(Fig. 5B)*. Another mechanism concerns temporary inactivation of P-TEFb, since only the activated form of the protein (bearing the phosphorylated T186 residue in the so-called T-loop of the CDK9) can interact with 7SK snRNP. Serine-threonine phosphatases are responsible for this process, including PPM1G, attracted by the NF-κB. Some proteins may also acetylate CycT, phosphorylate HEXIM, demethylate 7SK snRNA at the 5'-end, or carry out MePCE proteolysis, which leads to a destabilization of the complex and dissociation of P-TEFb. After the release from 7SK snRNP, the factor is again modified [52]. Let us note that the bulk of the P-TEFb that forms a complex with 7SK snRNP is associated with chromatin (e.g., through the Brd4 protein, interacting with the acylated histones H3 and H4), and the described mechanisms are often realized co-transcriptionally. Brd4 can also bind to P-TEFb in its complex with 7SK snRNP and initiate conformational changes in CycT and dissociation of CDK9 [53].

The best known mechanism of P-TEFb dissociation from its complex with 7SK snRNP is represented by TAR RNA in HIV-infected cells *(Fig. 5B)*. TAR RNA is a 5'-terminal structural element (hairpin) of the nascent strand of viral RNA synthesized from 5'-LTR. In the absence of additional activation, RNAP II is incapable of synthesizing transcripts longer than 60-80 nt from 5'-LTR, and TAR RNA consisting of 59 nt is the smallest fragment, followed by a transcription pause. In order to stimulate elongation, TAR RNA binds to a viral protein, Tat, which attracts various transcription factors to 5'-LTR, including P-TEFb [54]. This interaction is a result of specific contacts between the arginine-rich RNA-binding domain (ARM) of Tat and the trinucleotide side loop 5'-UCU-3' in the TAR RNA. At the same time, the apical loop of TAR RNA and the flanking region are associated with CycT *(Fig. 5B)*. This region of the molecule mimics the 5'-terminal hairpin of the 7SK snRNA, which enables TAR RNA binding to the ARM HEXIM, thus preventing the activation of P-TEFb in the absence of Tat. Moreover, Tat directly interacts with CycT and CDK9, forming a stable complex: that crystal structure was resolved in 2010 [55]. When binding to the so-called T-loop of CDK9, Tat changes the substrate specificity of the kinase, which then phosphorylates not only Ser2 in CTD of the Rpb1, but also Ser5 residues [56]. This allows HIV to activate transcription elongation even without the involvement of TFIIH *(Fig. 5B)*. Formation of a Tat-TAR-P-TEFb ternary complex is regulated by a number of enzymes that perform the acetylation, phosphorylation, methylation, and ubiquitination of Tat [54].

Obviously, there should be competition between the Tat-TAR RNA and 7SK snRNP complexes for binding to P-TEFb. As it turns out, Tat can displace the elongation factor from its complex with 7SK snRNP due to a direct interaction between Tat and CycT and conformational changes in P-TEFb [57, 58]. A similar mechanism was described for the RNA-binding proteins SRSF1 and SRSF2 that are involved in RNA splicing and metabolism in mammalian cells and are usually associated with the promoter regions of actively transcribed genes. Both proteins are capable of binding the 5'-terminal hairpin of 7SK snRNA, forming an alternative 7SK snRNP. If the nascent RNA stand contains the ESE sequence (exonic-splicing enhancer), SRSF1 and SRSF2 bind to it, resulting in a release of the active P-TEFb in close vicinity of RNAP II and the stimulation of transcription elongation of the required gene [59].

## NONCODING RNAs INTERACTING WITH OTHER TRANSCRIPTION FACTORS

**SRA RNA**

Human SRA RNA (steroid receptor activator) is a long ncRNA involved in the activation of estrogen (ER), progesterone (PR), glucocorticoid (GR), and other nuclear receptors. Similarly to 7SK snRNA, SRA RNA serves as a platform for the binding (including competitive) of various transcription factors. CTCF, SLIRP, and SHARP, as well as the RNA helicases p68 and p72, are the most important among them [60, 61]. Besides, SRA RNA modulates the activity of the transcription factor MyoD, which plays a key role in the differentiation of muscle cells [62]. SRA RNA is present in all human tissues, although its highest level is observed in the liver, heart, and skeletal muscles [63]. SRA RNA expression increases in females with polycystic ovarian and breast cancer, which justifies the rising interest in this RNA as a therapeutic target [61].

**Fig. 6:**
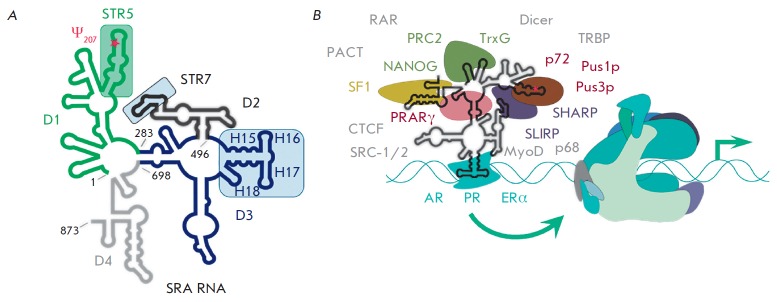
Schematic representation of the secondary structure of human SRA RNA (**A**) and its currently known protein partners (**B**) according to Liu *et al. *[61]. SRA RNA domains are colored: D1 – green, D2 – black, D3 – blue, D4 – grey. The U207 residue subjected to pseudouridilation is marked by an asterisk. Panel **A **shows the main structural elements of SRA RNA that bind several proteins, shown in color frames. Panel **B **shows a schematic representation of the proteins which directly bind to SRA RNA (they are labeled in corresponding colors). Nuclear receptors (AR – androgen, PR – progesterone, ERα – estrogen α) are colored in blue. All other proteins known to interact with SRA RNA are denoted in grey (without animation).

The *sra1* gene encoding SRA RNA is highly conserved in the genome of mice, rats, and humans. It is about 6,500 bp in length and consists of five exons. At least 20 different isoforms of SRA RNA, from 700 to 1,500 nt, have been detected in human cells. Most transcripts contain a core element of 687 nt, which corresponds to exons 2–5, and differ in their 5'- and 3'-end regions [64]. In 2012, chemical and enzymatic probing studies revealed the secondary structure of the 873- nt variant of RNA SRA, consisting of 25 hairpins (H1– H25) characterized by various lengths and shapes, which were conventionally divided into four domains, D1–D4, and 12 main structural elements, STR1–STR12 *(Fig. 6A)* [65]. Analysis of the deletion mutants of SRA RNA determined the six most important STRs that are responsible for the binding to certain proteins. Furthermore, removal of any STR results in complete or partial loss of the molecule’s functional properties; i.e., all of the main interactions occur owing to the multiplet structure of SRA RNA [66].

Nevertheless, the D3 domain (494–699 nt) is the most important one for the interaction with nuclear receptors. Its constituent element H15–H18 (505–575 nt) is highly conserved in vertebrates. Interestingly, individual expression of this element leads, on the contrary, to the inhibition of the transcription of ERα-dependent genes [67]. Switching of the SRA RNA function from activation to repression of nuclear receptors was also observed after replacement of the extremely important U207 residue in STR5 by adenosine. U207 is a site of pseudouridilation, which is carried out by the pseudouridine-synthetases Pus1p and Pus3p, coactivators of nuclear receptors. For example, direct interaction between SRA RNA and Pus1p in murine cells activates the transcription of genes dependent on retinoic acid receptors (mRARc) [68]. Synthetic oligonucleotide identical to STR5 can compete with full-length SRA RNA and block Pus1p, preventing a modification of this ncRNA, which results in the inhibition of the transcription of AR- and ERα-dependent genes [69].

STR7 of SRA RNA interacts with the RNA-recognition motifs (RRM) of the factors SHARP and SLIRP and, thereby, initiates both activation and inhibition of the transcription of various genes. Another SRA RNA partner, the receptor PPARγ, regulates the expression of the genes involved in the control of adipogenesis and insulin sensitivity [60]. Clearly, SRA RNA is one of the most important components among those engaged in the control of the activity of nuclear receptors, and it participates in various regulatory mechanisms, frequently accompanied by protein cascades (*Fig. 6B*).

Similarly to other ncRNAs, some SRA RNA suppression mechanisms exist that in particular involve the SRAP protein. The latter is encoded in 39% of SRA RNA transcripts and represents an example of specific self-regulation. In fact, SRA RNA is a coding RNA, although the key function is fulfilled by a non-coding variant of the *sra1 *gene transcript. At the moment, it remains unclear whether SRAP is capable of direct binding to SRA RNA and inhibition of its interaction with other proteins, or whether this process is carried out through the transcription factors or nuclear receptors associated with this RNA [70]. Nevertheless, the ratio between the amount of translated and untranslated products of *sra1* gene transcription is one of the key factors of transcriptional regulation in the cell. For example, in the case of myocyte differentiation, equilibrium is strongly shifted towards the non-coding SRA RNA and it can smoothly interact with transcriptional activators, attracting them to the MyoD-dependent promoter and activating the transcription of the respective genes [71].

**GAS5 RNA**

**Fig. 7:**
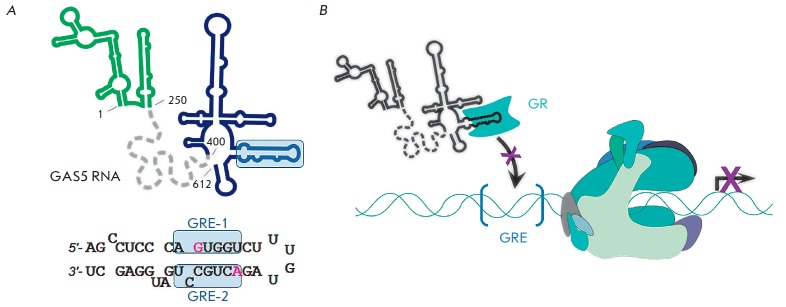
Inhibition of the transcription of glucocorticoid-dependent genes by GAS5 RNA. (**A**) Predicted GAS5 RNA secondary structure (top) and nucleotide sequence of the functional element (hairpin), containing the GRE-1 and GRE-2 regions that mimic DNA promoter (bottom). The key residues G540 and C554 are shown in pink. The GAS5 RNA domain responsible for the interaction with the HCV NS3 protein is colored in green. Central part of the molecule which is a target for miR binding is shown as a grey dash line (secondary structure is unknown). Both domains apparently do not participate in transcription regulation. (**B**) GAS5 RNA inhibits transcription by binding nuclear receptors and preventing their interaction with GRE-containing promoters.

GAS5 RNA (growth arrest-specific 5) is another long ncRNA that controls transcription through regulation of nuclear receptors. Under normal conditions, GAS5 RNA is rapidly degraded. However, in case of serum starvation in cells, arrested at a certain stage of growth, or after treatment with translation inhibitors, expression of GAS5 RNA is induced and its stability enhances, that gave the name for this ncRNA [72]. The main function of GAS5 RNA is to inhibit glucocorticoid receptor GR, the transcription factor responsible for activation of glucocorticoid genes. GR is a DNA-binding protein that recognizes the nucleotide sequence of GRE (gluticorticoid responsive element) in the promoter regions of controlled genes. The functional region of GAS5 RNA mimics GRE and, when binding to GR, blocks its access to promoters, thereby preventing activation of their transcription *(Fig. 7)*. GAS5 RNA can interact not only with GR, but also with other nuclear receptors binding GRE, in particular androgen, progesterone receptors, etc. [73]. Recent *in vitro *and *in vivo *studies clearly indicate the important role of GAS5 RNA in the initiation of apoptosis in various types of tumor cells and inhibition of proliferation and metastasis, as well as in immune response regulation in various inflammatory, bacterial, and viral diseases [74, 75].

Human GAS5 RNA is encoded by the *gas5 *gene*, *which comprises 12 exons interspersed with 10 introns encoding small nucleolar RNAs. After transcription performed by RNAP II, pre-mRNA undergoes polyadenylation and alternative splicing, which results in different isoforms of GAS5 RNA. The most important of them are GAS5a (612 nt) and GAS5b (651 nt) RNAs, containing 7a or 7b exons, respectively. Longer variants of GAS5 RNA (~1200–1800 nt) are less common and contain one or more sequences encoding small nucleolar RNAs [72, 76]. Under normal conditions, GAS5 RNA is localized in the cytoplasm and remains associated with the ribosome. However, in the case of arrested cell growth, it is translocated to the nucleus, where it interacts with the GR receptor [76].

The GAS5 RNA secondary structure is represented by several hairpins. The functional region (identified by deletion analysis) is located at the 3'-terminal region of the molecule (400–598 nt, *Fig. 7A*) and is found in all GAS5 RNA isoforms. The main contacts are formed between the GR and the GRE-1/ GRE-2 hairpin stem (539–544 and 553–559 nt), which mimic the conformation of the palindromic GRE-sequence of DNA: d(5'-AGAACANNNTGTTCT-3'/ 3'-TCTTGTNNNACAAGA-5'), where N = A, T, C, G). The G540 and C554 residues in GAS5 RNA are conserved among the human consensus GRE-sequences and interact with K442 and R447 in the DNA-binding domain of the GR protein, respectively. C554U substitution in GAS5 RNA, maintaining the stability of the double helix, results in a lost of the ability of this RNA to inhibit GR-dependent transcription from the MMTV (mouse mammary tumor virus) promoter *in vivo *[76]. Thus, GAS5 RNA competes with GRE-containing promoters for binding to GR similarly to bacterial 6S RNA, which also mimics the promoter and inhibits RNAP [36]. It has been shown that transfection of tumor cell lines with oligodeoxyribonucleotides identical to the 538–560 nt region of GAS5 RNA leads to effective apoptosis induction and a decrease of the cell survival rate [77], which could possibly enable using them for therapeutic purposes in the future.

The amount of GAS5 RNA in the cell is regulated by the NMD (nonsense-mediated RNA decay) system implementing the degradation of “nonsense” mRNA sequences and the mTOR (mammalian target of rapamycin) kinase-dependent signaling pathway. It is assumed that during active cell growth, mTOR-dependent translation of the short open reading frame (ORF) located in GAS5 RNA can occur (let us recall that this ncRNA is associated with the ribosome under these conditions). However, the large number of stop codons in the ORF and the short length of the potentially synthesized peptide lead to the activation of NMD and degradation of GAS5 RNA. In the case of cellular arrest and low level of the mTOR complex, GAS5 RNA is not translated and its concentration increases [73].

In addition to its primary function, GAS5 RNA binds oncogenic miR-21, miR-222, and miR-103, thereby serving as a siRNA-sponge and preventing them from impacting gene expression [74, 78]. Moreover, recent studies have shown that the 5'-terminal part of GAS5 RNA (1–250 nt) can bind the NS3 protein of the hepatitis C virus (HCV) and inhibit its function, thereby repressing HCV replication [79]. Obviously, multifunctionality of GAS5 RNA is achieved via different domains of the molecule: each one is responsible for interaction with a certain target *(Fig. 7)*.

## OTHER RNAS INVOLVED IN THE REGULATION OF TRANSCRIPTION FACTOR ACTIVITY

Dozens of ncRNAs modulating the activity of transcription factors are currently known. Most of them “work” only under certain (stress) conditions and are often tissue-specific [13, 17]. For example, NRSE RNA (neuron-restrictive silencer element) is expressed in stem cells and associated with the transcriptional repressor NRSF/REST responsible for neuron-specific gene silencing. The mechanism of this RNA-protein interaction is similar to the GAS5 RNA functional mechanism: NRSE RNA is a short (~20 bp) double stranded RNA that mimics the structure of the promoter. The NRSE-bound NRSF/REST factor turns into a transcription activator and “switches on” neuron-specific gene expression [80]. TSU RNA (trophoblast STAT utron), the 5'-untranslated end of the mRNA of the gene encoding the transcription factor STAT1, binds to its own protein and, thus, mimics the STAT-binding promoter and thereby inhibits gene expression of the major histocompatibility complex [81]. Long noncoding HSR1 RNA (~600 nt) activates HSF1, the main heat shock transcription factor, which initiates the functioning of the RNAP II elongation complex retained at stress gene promoters [82]. Other ncRNAs affect the transcription factor activity, altering their cellular localization; e.g., NRON RNA and lncRNA-p21 [83].

Special attention should be paid to circular RNAs (circRNAs or ciRNAs), products of alternative splicing, which results in closure of the 5'- and 3'-ends of the molecule. According to recent data, more than 100 circRNAs are associated with RNAP II and at least some of them activate transcription of their own genes [84]. It is assumed that circRNAs indirectly interact with RNAP II through the U1 snRNP of spliceosome, but the exact mechanism of their action is unknown [13].

## CONCLUSION

In recent years, increasingly detailed information has appeared regarding the various ncRNAs involved in transcription regulation in eukaryotic cells both at the level of specific genes and on a more global scale. Most often, they are long ncRNAs that regulate transcription during chromatin remodeling. In this review, we described those ncRNAs whose action mechanisms are closely related to the supervision of the functioning of the RNAP II transcription complex. The aforementioned ncRNAs have a number of other features which are equally important. For example, Alu RNA binds SRP9/14 (signal recognition particle) proteins and, as part of this RNP, inhibits translation initiation. In contrast, free Alu RNA is able to activate this process [85]. U1 snRNA is one of the main components of the spliceosome, and its participation in the activation of transcription factors is not that significant. At the same time, the ability of GAS5 RNA to interact with oncogenic miRs may be no less important than the ability to bind GR-receptors. Finally, it was shown that B2 RNA regulates transcription not only by interacting with RNAP II and inhibiting its activity, but also by direct binding to heat shock protein genes and inhibiting their expression in the absence of stress. Increased temperature results in the degradation of B2 RNA initiated by the EZH2 protein incorporated in the PRC2 complex and releases these genes for active transcription [86]. These and other facts attest to the diversity of the properties and functions of ncRNAs and undoubtedly demonstrate their importance in cell activity.

## Abbreviations

ncRNA: noncoding RNA
nt: nucleotide residues
PIC: preinitiating complex
RNAP: RNA polymerase
RNP: ribonucleoprotein
snRNA: small nuclear RNA
SINE: short interspersed elements
